# Multiunit Recording of Cerebellar Cortex in Autistic Male Rats during Social Interaction in Enriched Environments

**DOI:** 10.3390/neurosci4030016

**Published:** 2023-07-28

**Authors:** Omar E. Cruz-Magos, Grecia Herrera-Meza, Luis I. García, Genaro A. Coria-Avila, Deissy Herrera-Covarrubias, María Rebeca Toledo-Cárdenas, María Elena Hernández-Aguilar, Jorge Manzo

**Affiliations:** 1 Doctorado en Investigaciones Cerebrales, Universidad Veracruzana, Xalapa 91190, Veracruz, Mexico; 2 Benemérita Escuela Normal Veracruzana “Enríque C. Rébsamen”, Xalapa 91190, Veracruz, Mexico; 3 Instituto de Investigaciones Cerebrales, Universidad Veracruzana, Xalapa 91190, Veracruz, Mexico; luisgarcia@uv.mx (L.I.G.); gcoria@uv.mx (G.A.C.-A.); dherrera@uv.mx (D.H.-C.); rtoledo@uv.mx (M.R.T.-C.); elenahernandez@uv.mx (M.E.H.-A.)

**Keywords:** autism, valproic acid, cerebellum, hyperexcitability, socialization

## Abstract

Autism in humans is a lifelong behavioral disorder that typically manifests in early infancy, primarily affecting boys. It arises from neurodevelopmental changes that significantly impact social behavior, with the cerebellum being one of the principal affected regions. In this study, we investigated the cerebellum in an autism animal model, recording the multiunit activity of cerebellar vermis lobules 6 and 7 (L6 and L7) in male rats with autism-like behavior induced by postnatal valproate treatment. Two groups were formed: control (Ctrl) and experimental (VPA) males, which were further divided based on their living conditions into standard (Std) or enriched environments (EE). Social arenas were used for recording purposes. Both groups and lobules showed increased multiunit amplitude during social interaction (SI) and vertical exploration (VE), with higher amplitudes observed in VPA males. Interestingly, the EE significantly reduced the amplitude during SI, suggesting that EE promotes neural plasticity, resulting in improved social responses with fewer activated neurons, meaning improved activity with less energy consumption. Consequently, EE proves to be a valuable strategy for addressing the challenges associated with autism behavior.

## 1. Introduction

The cerebellum is a fascinating structure located caudal to both brain hemispheres and embracing the brainstem. It has a distinct shape, resembling a hemispherical ellipse, with a longer axis aligned in the coronal plane and a shorter axis in the sagittal plane [1]. Despite its importance, the cerebellum has often been overshadowed in research compared to the neocortex, leading to it being called the Cinderella of the central nervous system. However, accumulating evidence suggests that the cerebellum plays a crucial role in a wide range of functions, from motor control to cognition [2,3]. Moreover, studies have revealed that structural and functional changes in the cerebellum can underlie various disorders. For example, research has implicated the cerebellum in the autism spectrum disorder, with findings demonstrating anatomical and functional abnormalities in this central region [4,5]. This highlights the significance of the cerebellum not only in typical brain function but also in the understanding and potential treatment of neurodevelopmental conditions, such as autism spectrum disorder.

In humans, autism is a complex set of lifetime behavioral disorders that manifest in early infancy, primarily affecting boys, which is observed in a high prevalence in children worldwide. Extensive research is underway to uncover the neurophysiological alterations underlying autistic features, and one area of focus is the cerebellum, perhaps due to its protracted development, spanning from prenatal to postnatal periods, making it susceptible to various environmental factors that could potentially impact its function. Evidence in humans has revealed that the autistic cerebellum tends to exhibit a significant reduction (around 30–50%) in the number of Purkinje neurons in both the vermis and hemispheres, as well as fewer neurons in the fastigial and interpositus nuclei [4]. However, investigations using neuroimaging techniques have reported inconsistencies in the total volume of the cerebellum among autistic individuals, which could be attributed to variations in the characteristics of the subjects under study [5]. Regardless of the anatomical alterations, it is important to note that these structural differences often translate into functional abnormalities. For instance, electroencephalography studies showed power abnormalities in the autistic brain, along with local overconnectivity and long-range underconnectivity [6]. Moreover, the cerebellum has been found to exhibit decreased activation during attention-related tasks and increased activation during simple motor tasks [7,8]. Therefore, investigating the activity of the central nervous system, with a specific emphasis on the cerebellum, is crucial for gaining a deeper understanding of the neurobiology of autism.

To contribute to this knowledge, the present study aimed to record the multiunit activity of lobules 6 and 7 in the vermis of the cerebellum in rats with induced autism. The focus of the investigation was to record the lobules during the observation of the social behavior of male rats and assess the impact of environmental stimulation through an enriched environment.

## 2. Materials and Methods

### 2.1. Subjects

Pregnant Wistar female rats were obtained from the animal colony of our institute, where they were maintained in a 12 h reversed light-dark cycle, with the light turning off at 0800, and kept alone in a single cage to ensure health and well-being. After birth, male pups were randomly assigned to either experimental or control groups. The experimental group represented the postnatal autistic model. From postnatal days 6 to 12, experimental pups received a daily intraperitoneal injection of valproic acid (VPA) diluted in a physiological saline solution. As previously described, the dosage administered was 150 mg/kg of body weight [9], resulting in a mortality rate of less than 10 percent. The Control group consisted of males that received only the physiological saline solution injected in the same volume as the experimental group (1 mL/kg). Weaning of the pups was on postnatal day 21, after which the experimental and control males were housed together until the implantation of recording electrodes. All the protocols and procedures conducted in this study were approved by the local animal care committee (CICUAL) of the Institute for Brain Research at Universidad Veracruzana, Mexico. The study followed the guidelines and regulations outlined in the Official Mexican Norms (NOM-062-Z00-1999 and NOM-087-ECOL-SSA1-2003) and the Society for Neuroscience Policy on the Use of Animals in Neuroscience Research.

### 2.2. Electrodes Implantation

On postnatal day 47, the animals underwent the procedure for electrode implantation. They were first anesthetized using an intraperitoneal injection of a 2:1 mixture of ketamine (50 mg/kg): xylazine (10 mg/kg). The animals were then secured onto a stereotaxic frame (Stoelting, Co., Wood Dale, IL, USA). A stainless steel monopolar recording electrode (FHC, Inc., Bowdoin, ME, USA) with a tip size of 250 µm and a resistance of 3 MΩ was implanted into the cerebellar lobule 6 or 7. A stainless steel screw was also implanted in the skull as the reference electrode. The coordinates for placing the recording electrode tip were determined using the Paxinos and Watson stereotaxic atlas [10]. The coordinates for lobule 6a relative to Bregma were as follows: Anteroposterior −12.5 mm, Lateral −0.1 mm, and Dorsoventral 1.6 mm; for lobule 7, respectively were −15.2 mm, −0.1 mm, and 3.4 mm. Post-surgical treatment for analgesia and antibacterial purposes consisted, respectively of three days of subcutaneous injections of flunixin meglumine (2.5 mg/kg) and enrofloxacine (5 mg/kg). Recordings were conducted while the animals were in free movement in the socialization arena (see below). The recording and reference electrodes were connected to a Grass 15LT amplifier system (Astro-Med, Inc., West Warwick, RI, USA). The system generated duplicated signals, sending one to an audio monitor (Grass AM9) and the other to a PC computer equipped with the PolyVIEW Data Acquisition and Analysis Software (PVA-16, Astro-Med, Inc., USA). Although the saved file contained the responses throughout the entire behavioral period, representative traces of 2 s each, corresponding to social interaction or vertical exploration, were randomly selected. The maximum amplitude observed during the 5–8 selected traces per animal for each specific behavior was used for statistical comparisons and graphical representation. After completing the behavioral test, the animals were deeply anesthetized using intraperitoneal sodium pentobarbital (60 mg/kg). They were then placed back into the stereotaxic frame, and the recording electrode was connected to a Grass CCU1 Constant Current Unit, driven by a Grass S48 stimulator (Astro. Med, Inc., USA). A constant current of 50 µA for 25 s was applied to induce an electrolytic lesion, allowing precise determination of the recording area. The data presented in the Section 3 only includes animals for which the electrode tip was correctly positioned within the desired location.

### 2.3. Enriched Environment and Recording Procedure

During the experimental period, specifically on postnatal days 50–59, the animals were divided into two groups (n = 6 each) depending on the environment of the behavioral arena (100 × 100 × 40 cm). One group was placed in an environment with chip wood bedding called the plain or Standard (Std) environment. The other group was set in an enriched environment (EE) arena designed to provide additional sensory and motor stimulation. The EE arena included various structures and items that encouraged movement and engaged multiple senses. These stimuli had textured surfaces, odors from inaccessible fruits, and a jingle bell sound. Each day, the animals were placed in their respective arena for 2 h, between 12:00 p.m. and 16:00 p.m. On postnatal day 60, the recordings of the vermis lobules began. At this stage, the animals were allowed to move freely within a socialization arena measuring 60 × 40 × 22 cm. The recording sessions commenced with a five-minute period where the subjects were alone in the socialization arena. Following the solitary period, a small wire cage containing a novel male animal was introduced and placed in one corner of the arena. The presence of the male served as a stimulus for social interaction. During this phase, vermis recordings were conducted for 10 min, and the data obtained were saved for analysis. The recordings were marked and categorized based on two specific behaviors observed in the arena. The first behavior was Vertical Exploration, which involved the male raising onto the hind legs. The second behavior was Social Interaction, characterized by the male’s nose poking through the novel male wire cage grid.

### 2.4. Statistics

The data pertaining to the amplitude of recordings are reported as means ± SEM. Statistical analysis and graph generation were performed using Prism 9.5 software (GraphPad, San Diego, CA, USA). A One-Way ANOVA was conducted to assess the significance of differences in recording amplitudes. Subsequently, Dunnet’s Multiple Comparisons Test was employed to determine specific pairwise comparisons. Additionally, *t*-tests were done to compare Ctrl vs. VPA data. Significance was inferred when *p* < 0.05.

## 3. Results

**Figure 1:**
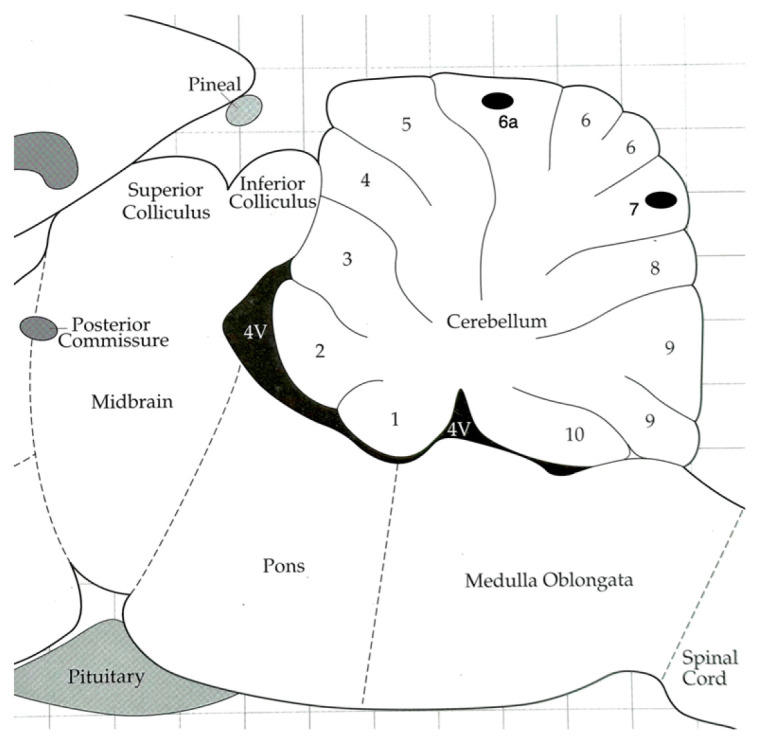
Sagittal section of the cerebellar vermis. Dots show the approximate area where the tip of the electrodes was placed for recording in lobules 6a and 7. The image was modified from that found in the Paxinos atlas [10].

The implantation procedure for the electrodes was performed with great success in most animals, resulting in accurately positioned electrodes for reliable data acquisition. Additionally, reference electrodes were appropriately implanted in the skull. Figure 1 visually depicts the approximate placement of the electrode tips within the superficial region of Lobule 6a and Lobule 7. Data represent targeted recordings from these specific cerebellar regions.

**Figure 2:**
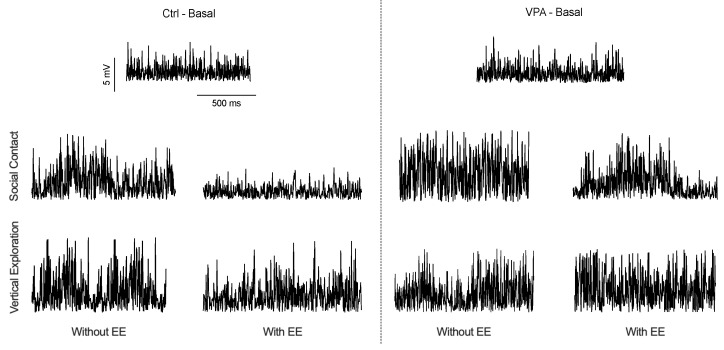
Amplitude of multiunit recordings at the cerebellar cortex. The figure displays upward representative traces for lobules 6 and 7. The basal trace represents the multiunit activity observed when the male moves alone in the experimental cage. After introducing a second subject, the multiunit amplitude undergoes changes depending on the environmental context. Variations in amplitude are mainly observed when the experimental male interacts with the introduced subject.

Figure 2 visually represents the recorded data observed on the computer screen, showcasing three distinct recording scenarios: basal, vertical exploration, and social interaction. It is important to note that while the discharge frequency remained unchanged, noticeable variations in recording amplitudes were observed corresponding to the behavioral situations. During the basal recording, both control (Ctrl) and valproic acid (VPA) male subjects showed similar low amplitude records while alone in the chamber. Following social interaction in the Std environment, discharge amplitude increased in Ctrl and VPA males. However, this increased amplitude was observed to be lower when the Ctrl males were exposed to an EE. Similarly, VPA males in the EE also exhibited a decreased amplitude but with a lower magnitude. Notably, no significant changes in the recorded amplitudes were observed during vertical exploration, and no further analyses were done.

**Figure 3:**
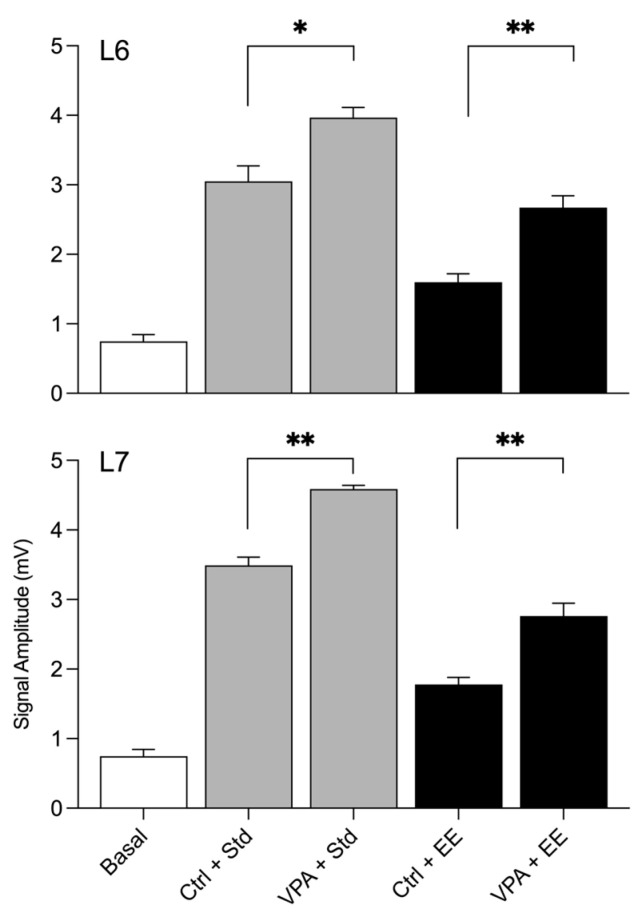
Multiunit amplitude during social interactions. The basal bar shows the amplitude of the experimental male before introducing the social partner. The interaction with the second subject in the arena significantly increased the amplitude in all groups (asterisks for differences are not shown). The *t*-test significant asterisks show that Ctrl and VPA males had different amplitudes. Additionally, bars show a reliable difference between males from Std and EE conditions. * = *p* < 0.05; ** = *p* < 0.01.

The statistical results of the amplitudes during social interactions are depicted in the graphical representation shown in Figure 3. By comparing the basal amplitudes with those obtained in the social arena of Std environment subjects, it was found that the Ctrl and VPA groups exhibited a significant increase in amplitude in both lobule 6 and lobule 7. However, an interesting effect was observed when both groups were exposed to the EE. The impact of the EE environment led to a low amplitude recorded in both lobule 6 and lobule 7.

## 4. Discussion

The cerebellum, a prominent structure in the central nervous system, plays a significant role in various functions. Its intricate arrangement of neurons and glia makes it remarkably precise yet highly sensitive. Extensive research has demonstrated the cerebellum’s sensitivity to substances such as alcohol [11], cannabis [12], nicotine [13], and other drugs of abuse [14]. In addition to these substances, the present study revealed that the cerebellum is also responsive to the postnatal administered valproic acid (VPA), a commonly used medication. Interestingly, our results indicate that VPA modifies the electrical activity in the cerebellar cortex, summing in turn to the emergence of autism-like behaviors. This finding highlights the potential role of the cerebellum in the development and manifestation of autism spectrum disorder in males. Notwithstanding, it is important to acknowledge that the present study primarily focuses on two specific cerebellar lobules of the vermis and a specific spot of the whole lobule. While these findings provide valuable insights into the neural activity and behavioral responses within these particular lobules, it is crucial to recognize the broader complex nature of the cerebellum as a whole, as we showed previously [15].

Lobules 6 and 7 of the cerebellum are characterized by a baseline multiunit discharge with low amplitude. This basal activity amplifies when control (Ctrl) or valproic acid (VPA) male subjects engage in social contact or vertical exploration behaviors, although the neural activity observed in VPA males exhibits a significantly higher level of amplification during social interaction. The increased amplitude suggests the recruitment of a larger number of neurons, which appears to be a necessary response to trigger specific behaviors. Similar neural responses have been observed in other central nervous system regions during approaching or contact behavior [16] and during sexual behavior [15]. These findings suggest a shared mechanism of neural activation across different brain regions underlying the expression of specific behaviors. However, it is crucial to consider that not all cerebellum regions exhibit equal responses. As demonstrated in our previous study [17], the cerebellar fastigial nucleus, for instance, displays a distinct mirror-like response, particularly in the context of evoking sexual behavior. This highlights the regional specificity and functional diversity within the cerebellum, underscoring the importance of considering different cerebellar areas and their unique contributions to behavior.

The findings from our study, as previously mentioned, revealed that VPA males exhibited a significantly higher amplitude in the multiunit response, suggesting the activation of a greater number of neurons surrounding the recording electrode. This heightened neural response may be attributed to the hyperactivity of the cortex in the recorded lobules, aligning with the notion of hyperfunctioning local microcircuits in autism [18]. Existing data from other studies have also provided insights into the hyperexcitability response observed in autism. Research on adult rats lacking the CNTNAP2 gene, associated with language-related disorders such as autism, demonstrated hyperexcitability in the auditory cortex [19]. This supports the notion that hyperresponsiveness within cortical circuits may contribute to the manifestation of autism-related characteristics. Furthermore, investigations using a mouse model of autism have also reported hyperfunctioning of granule cells in the cerebellum [20]. These findings corroborate our results and the accumulating evidence in the literature, collectively pointing toward the cortex cerebellum’s tendency to become overreactive in individuals with autism.

The impact of the enriched environment (EE) on the recording amplitude of lobules 6 and 7 of the cerebellum is another notable finding in our study. Both the Ctrl and VPA males exhibited social behavior, albeit with a reduced amplitude at the recording sites. This suggests that the EE refines the neural circuitry to promote efficient behavioral responses with fewer activated neurons, notwithstanding that in VPA subjects, it still does not promote Ctrl values. However, this result highlights the potent efficacy of EE as a strategy to enhance the function of the cerebellum in individuals with autism. It aligns with other studies that have demonstrated the effectiveness of EE in promoting brain plasticity [21]. Moreover, our previous work has shown the benefits of EE in modulating molecular characteristics in the cerebellar cortex of mice [22], improving reproductive behavior in zebrafish [23], and enhancing cutaneous sensitivity in children with autism [24]. EE also exerts a similar impact to acquiring behavioral experience, as we have previously demonstrated in typical subjects, where gaining expertise leads to a reduced amplitude in multiunit recordings alongside the execution of efficient behaviors [1,17]. In essence, EE proves to be a valuable strategy for addressing the behavioral challenges associated with autism. However, further studies are necessary to explore and refine specific EE procedures that can serve as therapeutic interventions to modulate brain circuits more effectively.

## 5. Conclusions

The valproic acid model of autism in rats is widely used to study the neurobiological basis of the disorder. In our study, we investigated the multiunit activity of cerebellar lobules 6 and 7 in the postnatal VPA model and observed hyperactivity in these regions. However, our study concluded that using an enriched environment (EE) produces relief in the characteristics of the recording amplitude. This suggests that EE has the potential to modulate the neural activity in the cerebellum and alleviate the hyperactivity circuits in autism. Nevertheless, further research is needed to fully understand the specific mechanisms through which EE exerts its effects and to establish customized EE protocols that can serve as effective therapeutic interventions for individuals with autism.

## Data Availability

Data unavailable due to privacy.
